# Supplementary material for: Whole-genome sequence analyses reveal cryptic species-level diversity among clinical Actinotignum isolates misidentified by matrix-assisted laser desorption/ionization time-of-flight mass spectrometry
Source: Microbiol Spectr. 2026 Feb 24;14(4):e02190-25. doi: 10.1128/spectrum.02190-25 (PMC13055207; doi:10.1128/spectrum.02190-25)
Supplement: Figure S1 — Core genome phylogeny. [file spectrum.02190-25-s0001.pdf]

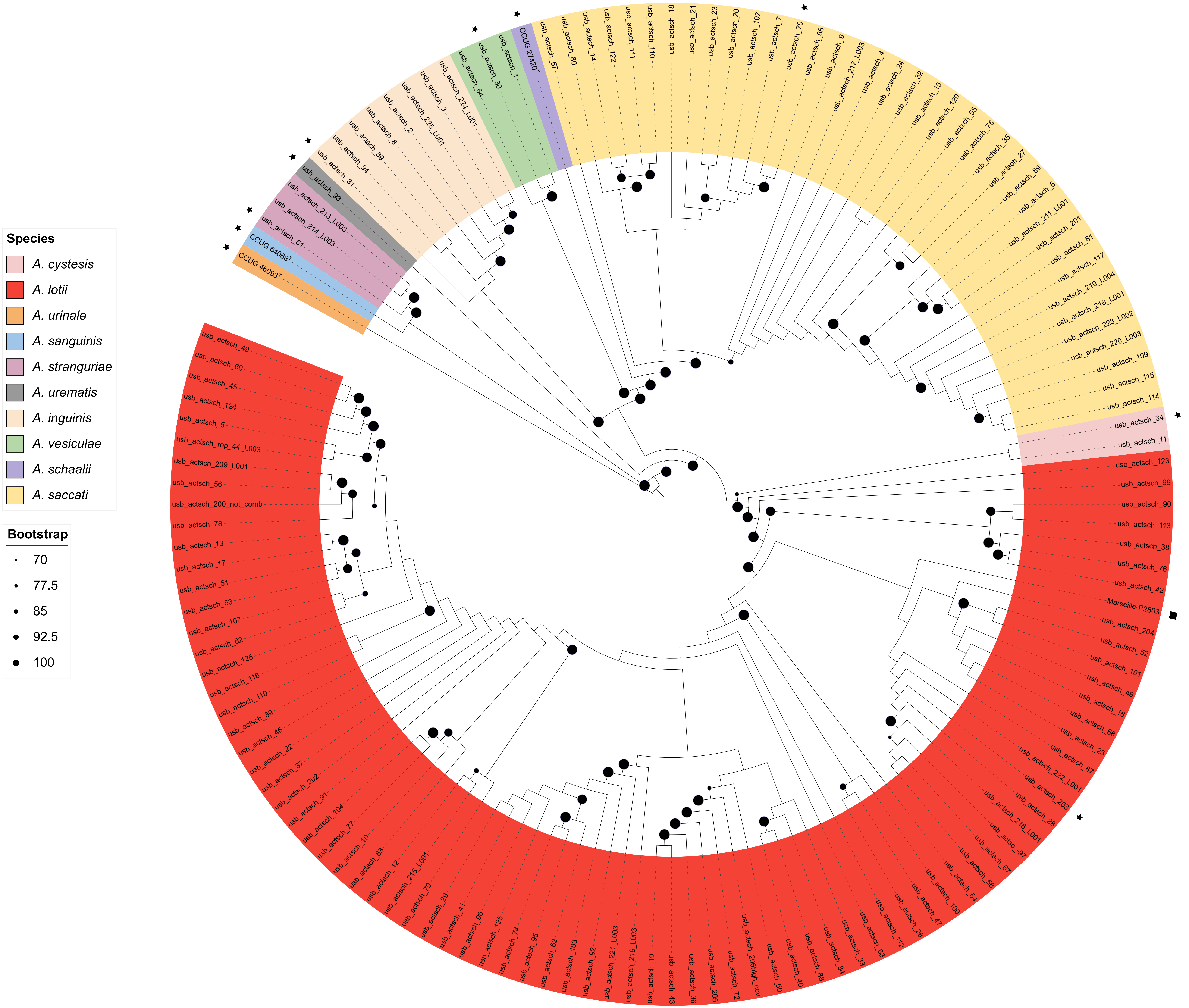
Species
A. cystesis
A. lotii
A. urinale
A. sanguinis
A. stranguriae
A. urematis
A. inguinis
A. vesiculae
A. schaalii
A. saccati
Bootstrap
70
77.5
85
92.5
100
CCUG 46093ᵀ
CCUG 64068ᵀ
usb_actsch_61
usb_actsch_214_L003
usb_actsch_213_L003
usb_actsch_93
usb_actsch_31
usb_actsch_94
usb_actsch_89
usb_actsch_8
usb_actsch_2
usb_actsch_3
usb_actsch_225_L001
usb_actsch_224_L001
usb_actsch_64
usb_actsch_30
usb_actsch_1
CCUG 27420ᵀ
usb_actsch_57
usb_actsch_80
usb_actsch_14
usb_actsch_122
usb_actsch_111
usb_actsch_110
usb_actsch_18
usb_actsch_21
usb_actsch_23
usb_actsch_20
usb_actsch_102
usb_actsch_7
usb_actsch_70
usb_actsch_65
usb_actsch_9
usb_actsch_217_L003
usb_actsch_4
usb_actsch_24
usb_actsch_32
usb_actsch_15
usb_actsch_120
usb_actsch_55
usb_actsch_75
usb_actsch_35
usb_actsch_27
usb_actsch_59
usb_actsch_6
usb_actsch_211_L001
usb_actsch_201
usb_actsch_81
usb_actsch_117
usb_actsch_210_L004
usb_actsch_218_L001
usb_actsch_223_L002
usb_actsch_220_L003
usb_actsch_109
usb_actsch_115
usb_actsch_114
usb_actsch_34
usb_actsch_11
usb_actsch_123
usb_actsch_99
usb_actsch_90
usb_actsch_113
usb_actsch_38
usb_actsch_76
usb_actsch_42
Marseille-P2803
usb_actsch_204
usb_actsch_52
usb_actsch_101
usb_actsch_48
usb_actsch_16
usb_actsch_68
usb_actsch_25
usb_actsch_87
usb_actsch_222_L001
usb_actsch_203
usb_actsch_28
usb_actsch_216_L001
usb_actsc_97
usb_actsch_67
usb_actsch_58
usb_actsch_54
usb_actsch_100
usb_actsch_47
usb_actsch_26
usb_actsch_112
usb_actsch_63
usb_actsch_33
usb_actsch_84
usb_actsch_88
usb_actsch_40
usb_actsch_50
usb_actsch_206high_cov
usb_actsch_72
usb_actsch_205
usb_actsch_36
usb_actsch_43
usb_actsch_19
usb_actsch_219_L003
usb_actsch_221_L003
usb_actsch_92
usb_actsch_103
usb_actsch_62
usb_actsch_95
usb_actsch_74
usb_actsch_125
usb_actsch_96
usb_actsch_41
usb_actsch_29
usb_actsch_79
usb_actsch_215_L001
usb_actsch_12
usb_actsch_83
usb_actsch_10
usb_actsch_77
usb_actsch_104
usb_actsch_91
usb_actsch_202
usb_actsch_37
usb_actsch_22
usb_actsch_46
usb_actsch_39
usb_actsch_119
usb_actsch_116
usb_actsch_126
usb_actsch_82
usb_actsch_107
usb_actsch_53
usb_actsch_51
usb_actsch_17
usb_actsch_13
usb_actsch_78
usb_actsch_200_not_comb
usb_actsch_56
usb_actsch_209_L001
usb_actsch_rep_44_L003
usb_actsch_5
usb_actsch_124
usb_actsch_45
usb_actsch_60
usb_actsch_49
