# Supplementary material for: Whole-genome sequence analyses reveal cryptic species-level diversity among clinical Actinotignum isolates misidentified by matrix-assisted laser desorption/ionization time-of-flight mass spectrometry
Source: Microbiol Spectr. 2026 Feb 24;14(4):e02190-25. doi: 10.1128/spectrum.02190-25 (PMC13055207; doi:10.1128/spectrum.02190-25)
Supplement: Supplemental legend — Descriptive legend for Fig. S1. [file spectrum.02190-25-s0002.docx]

**Supplementary Figure S1**. Core genome phylogeny based on the concatenated sequences of 378 core proteins of 137 novel *Actinotignum* genomes and the *A. schaalii*, *A. sanguinis* and *A. urinale* type strains. All type strains are marked with a star, while *“A. timonense”* strain Marseille-P2803 is marked with a square. The tree was constructed using the maximum likelihood method. Boostrap values of 100 replicates are indicated in the nodes, represented by circles, and range from 70% to 100 %. Members of each phylogenomic species are represented in the same color and the species are displayed in counterclockwise order.
